# Evaluation of single and multilocus DNA barcodes towards species delineation in complex tree genus *Terminalia*

**DOI:** 10.1371/journal.pone.0182836

**Published:** 2017-08-22

**Authors:** Priyanka Mishra, Amit Kumar, Akshitha Nagireddy, Ashutosh K. Shukla, Velusamy Sundaresan

**Affiliations:** 1 Plant Biology and Systematics, CSIR—Central Institute of Medicinal and Aromatic Plants, Research Center, Bengaluru, Karnataka, India; 2 Biotechnology Division, CSIR—Central Institute of Medicinal and Aromatic Plants, Lucknow, Uttar Pradesh, India; Istituto di Biologia e Biotecnologia Agraria Consiglio Nazionale delle Ricerche, ITALY

## Abstract

DNA barcoding is used as a universal tool for delimiting species boundaries in taxonomically challenging groups, with different plastid and nuclear regions (*rbcL*, *matK*, *ITS* and *psbA-trnH)* being recommended as primary DNA barcodes for plants. We evaluated the feasibility of using these regions in the species-rich genus *Terminalia*, which exhibits various overlapping morphotypes with pantropical distribution, owing to its complex taxonomy. *Terminalia bellerica* and *T*. *chebula* are ingredients of the famous *Ayurvedic Rasayana* formulation *Triphala*, used for detoxification and rejuvenation. High demand for extracted phytochemicals as well as the high trade value of several species renders mandatory the need for the correct identification of traded plant material. Three different analytical methods with single and multilocus barcoding regions were tested to develop a DNA barcode reference library from 222 individuals representing 41 *Terminalia* species. All the single barcodes tested had a lower discriminatory power than the multilocus regions, and the combination of *matK+ITS* had the highest resolution rate (94.44%). The average intra-specific variations (0.0188±0.0019) were less than the distance to the nearest neighbour (0.106±0.009) with *matK* and *ITS*. Distance-based Neighbour Joining analysis outperformed the character-based Maximum Parsimony method in the identification of traded species such as *T*. *arjuna*, *T*. *chebula and T*. *tomentosa*, which are prone to adulteration. *rbcL* was shown to be a highly conservative region with only 3.45% variability between all of the sequences. The recommended barcode combination, *rbcL*+*matK*, failed to perform in the genus *Terminalia*. Considering the complexity of resolution observed with single regions, the present study proposes the combination of *matK*+*ITS* as the most successful barcode in *Terminalia*.

## Introduction

The genus *Terminalia* Linn. (the second largest genus in the Combretaceae) comprises approximately 200 species of trees and shrubs [1, 2] with pantropical distribution [3, 4]. In India, nearly 24 species have been reported from various regions [5]. The widely used species of *Terminalia* include *T*. *arjuna*, *T*. *bellerica*, *T*. *chebula* and *T*. *cattapa* and the whole plant is important for medicinal applications. Species of *Terminalia* possess diverse medicinal properties, viz. antioxidant, antidiabetic, antibacterial, antiviral, antifungal, anticancer, antiulcer, antimutagenic and wound healing activities and are an integral part of various *Ayurvedic* formulations sold under the trade names *Triphala* (a rejuvenator for the digestive tract and a mild laxative), *Haritaki*, *Arjuna*, *Baheda*, *Harad*, etc [6–9]. The fruits and bark of certain species of the genus are traded in large volumes (2000–10000 metric tons per year) [10]. The highly valuable species *T*. *arjuna* has been classified as “near–threatened” at the regional level, reinforcing the plans for its conservation (*envis*.*frlht*.*org / frlhtenvis*.*nic*.*in*). Moreover, Nair [11], through his book *Controversial Drug Plants*, reported cases of species substitution of *Terminalia* in genuine drugs. Due to the morphological similarity of *T*. *pallida* fruits with *T*. *chebula* fruits, and of *T*. *arjuna* bark with *T*. *tomentosa* bark, the species are prone to adulteration in trade markets.

The taxonomic status of *Terminalia* species has been very controversial as they exhibit various overlapping morphotypes scattered in diverse regions and vary considerably in morphology and karyotype. The presence of typical fruit features, flowering pattern, and nectar glands at the base of petiole caused its classification into sub-divisions or sections by some taxonomists, but no universal conclusion could be proposed [12–14]. Recent comprehensive studies of the phylogenetic framework of the family Combretaceae confirmed this complexity and proposed the revision of generic classification with well-resolved and supported results [4, 15]. Thus, the lack of sufficient taxonomical characters for accurate species identification requires the investigation of molecular gene pools that could be of use for the authenticated use of *Terminalia* species in herbal market and their resource protection. In addition, many *Terminalia* species are the source of high value timber and various non-wood forest products (NWFP) throughout the Indian sub-continent. Due to high exploitation by the pharmaceutical industry, large volume trade at national, international and regional levels, the losses incurred by over-grazing, indiscriminate felling of trees and conversion of forest land into agricultural land as well as human settlements, a rapid depletion of *Terminalia* genetic resources has been recorded. Thus, the genus *Terminalia* is seriously endangered and there is a need to conserve the remaining genetic diversity.

Taxonomical delimitation of species based on morphology has always been challenging, due to a certain degree of internal variation. Over the years, technological advances have provided us with much more robust identification tools, among which, DNA barcoding holds the promise of resolving the high level of complexity present in some taxonomic groups. DNA barcoding technology offers an alternative and feasible tool to catalogue biodiversity, by comparing standardized portions of the genome, universally present in the target lineages that contain sufficient nucleotide sequence variation to allow species identification. Suitable threshold between the inter- and intra-specific genetic distances, termed as DNA barcoding gap provides an independent method to define systematics relationships, resulting in a robust barcode [16, 17]. Besides its potential use as a robust taxonomic tool, DNA barcoding finds use in a range of applications including plant-based herbal product identification / authentication, forensic analysis, forest biodiversity assessment and ecological studies, as well as providing a cost-effective system for reliable identification of biological material involved in trade [18–21]. The 5´ end of cytochrome c oxidase 1 (*COX*1) from the mitochondrial genome is largely employed as a universal barcode marker in animals, but its lower mutation rate precludes its usage as a universal plant barcode [17, 22]. In contrast, the higher rate of evolution, lack of recombination and stable structure of the chloroplast genome makes it a good candidate for plant barcoding [23–25]. Significant progress during the last decades proposed several individual candidate regions such as *matK*, *rbcL*, *psbA-trnH*, *ITS*, *trnL*-*F*, *5S-rRNA* and *18S-rRNA* for use in plant species discrimination [23, 26–31]. This limitation has led to the development of a multilocus, combinatorial approach that makes use of different combinations of coding and noncoding plastid regions and nuclear ribosomal spacer genes [27, 31], as reported in several studies [21, 32–35].

Even so, inter-specific variation in large and complex genera may be problematic [36]. Against this background, our study was designed to evaluate the applicability of prevailing concepts to complex tree species. The work presented is the first attempt made for establishing *Terminalia* species barcode reference library by using both a single and a multi-locus approach. We used four of the primary DNA barcoding sequences viz. *rbcL*, *matK*, *psbA-trnH* and *ITS* to pursue the following objectives: (i) to assess the selected loci or their combinations as candidate DNA barcodes in species discrimination for the genus *Terminalia*, (ii) to compare the technical feasibility of different analytical methods on species resolution rates in tree species, and (iii) to examine the congruence of traditional phylogenetic framework with DNA barcoding results for closely related species.

## Materials and methods

### Taxon sampling

A total of 222 individuals of 41 *Terminalia* species were assembled for this study. For obtaining the sequences generated from experiments in our lab, a total of 33 individuals corresponding to 5 different species of *Terminalia* were examined from natural populations of India (S1 Table). The field samples represented all the putative species of *Terminalia* viz., *T*. *arjuna*, *T*. *bellerica*, *T*. *chebula*, *T*. *catappa*, and *T*. *paniculata*, which were reported to exist with various morphotypes scattered in diverse regions. *Anogeissus acuminate*, *Pteleopsis anisoptera* and *Bucida buceras* were selected as the out-group for tree-based analysis based on recent molecular evidence by Maurin et al. 2010 [4]. Healthy fresh leaves for all the individual samples were collected and desiccated in silica gel for DNA extraction. Vouchers for each species sampled for this study were deposited in the herbarium at CSIR—Central Institute of Medicinal and Aromatic Plants, Lucknow for future reference. Apart from the sequences obtained from our own laboratory samples, all of the nucleotide sequences belonging to the genus *Terminalia* for the tested regions were downloaded from the NCBI database. The sequences were filtered out on the basis of various criteria. Sequences with length < 300 bp, lack of voucher specimens and those categorised as unverified in GenBank were omitted. Thus database sequences from only genuinely identified species were included in the present study. An effort was made to include a minimum of five individuals for each species, but due to the scarcity of sequence data for certain species in the NCBI database and difficulties in obtaining these plants in the field, some species were represented with less than five individuals. In order to save computational time and avoid the problems associated with large data sets, the representation of each species was limited to between three and five.

### DNA extraction, PCR amplification and sequencing

Isolation of high quality DNA has proved to be a major bottleneck in *Terminalia* species due to presence of large amount of secondary metabolites [37]. Based on the published methods, we attempted the isolation of high quality genomic DNA from the leaf tissue of individual accessions using the cetyl trimethyl ammonium bromide (CTAB) protocol [38] with necessary modifications. The concentrations of β-mercaptoethanol and PVP (polyvinylpyrrolidone) were increased to 2% (v/v) and 4% (w/v), respectively. An additional chloroform-isoamyl alcohol (96:4) purification step was performed to remove proteins and potentially interfering secondary metabolites. Isolated DNA was checked for its quality and quantity by electrophoresis on a 0.8% agarose gel and spectrophotometric analysis (NanoDrop, ND-1000, USA), respectively. The DNA was diluted to a final concentration of 30–50 ng/μl for polymerase chain reaction (PCR) amplification. Two plastid barcodes (the coding genes *matK* and *rbcL*) and the nuclear ribosomal internal transcribed spacer (*ITS*) were amplified according to PCR reaction conditions following guidelines from the Consortium of Barcode of Life (CBOL) plant-working group, and sequenced using universal primers. The *psbA*-*trnH* intergenic spacer region was tested with two different primer sets: fwdPA and revTH [39] and psbA and trnH [23] corresponding to species range, but unsuccessful amplification led to its omission from the present study (Table 1). The selected DNA regions were amplified by using a standard PCR, whereby amplification for each primer set was carried out in a 50-μl volume solution containing 1X Taq DNA polymerase buffer, 200 μM each dNTP (dATP:dTTP:dCTP:dGTP in 1:1:1:1 parts), 10 pmol of each primer (forward and reverse), 1 unit of Taq DNA polymerase and 25 ng of template DNA. The PCR products were purified with a Nucleospin PCR purification kit using the manufacturer’s (MACHEREY-NAGEL– 07 / 2014, Rev.03) protocol. Sequencing reactions were performed using the BigDye Terminator v3.1 Cycle Sequencing Kit (Applied Biosystems, Foster City, CA) on an ABI 3130 XL automated sequencer (Applied Biosystems). All the laboratory protocols can be accessed online through http://dx.doi.org/10.17504/protocols.io.h4rb8v6.

**Table 1:** Details of primers and their amplification conditions used in the study.

Region	Primer	Sequence (5´-3´)	Thermocycling conditions	Reference
***ITS***	**ITS5a**	5'-CCTTATCATTTAGAGGAAGGAG-3'	94°C 5 min; [30 cycles: 94°C 1 min; 50°C 1 min; 72°C 1.5 min]; 72°C 7 min	[23]
	**ITS4**	5'-TCCTCCGCTTATTGATATGC-3'		
***rbcL***	rbcL1F	5'-ATGTCACCACAAACAGAAAC-3‘	95°C 2 min; [35 cycles: 94°C 1 min; 55°C 30 s; 72°C 1 min]; 72°C 7 min	[23]
	rbcL724R	5'-TCGCATGTACCTGCAGTAGC-3‘		
***matK***	matK 390F	5'-CGATCTATTCATTCAATATTTC-3’	95°C 2 min; [30 cycles: 94°C 1 min; 48°C 30 s; 72°C 1 min]; 72°C 7 min	[23]
	matK 1326R	5'-TCTAGCACACGAAAGTCGAAGT-3'		
***psbA-trnH***	fwdPA	5'-GTTATGCATGAACGTAATGCTC-3	94°C 5 min; [35 cycles: 94°C 1 min; 55°C 30 s; 72°C 1.5 min]; 72°C 7 min	[39]
	revTH	5'-CGCGCATGGTGGATTCACAATCC-3'		
	psbA	5'-GTTATGCATGAACGTAATGCT-3'	94°C 5 min; [35 cycles: 94°C 1 min; 55°C 30 s; 72°C 1.5 min]; 72°C 7 min	[23]
	trnH	5'-CGCGCATGGTGGATTCACAATCC-3'		

### Data analysis

The electropherograms obtained for each region were base-called using PHRED. Raw sequences obtained for each region were assembled and edited using CodonCode Aligner v.3.0.1 (CodonCode Corporation). Finally, the sequences were blasted on NCBI BLAST under the programme BLASTN 2.2.1+ and on to BOLD using Identification Request for checking their homology with other available sequences. Each barcode sequence was greater than 500bp in length and free from contamination. The edited sequences were then aligned with Muscle 3.8.31 on the EMBLEBI website (http://www.ebi.ac.uk) under default parameters and adjusted manually in BioEdit v7.1.3.0 [40]. All of the variable sites were rechecked on the original trace files. The nucleotide sequences corresponding to the regions *ITS*, *rbcL* and *matK* were analysed separately (*ITS*, *rbcL*, *matK*) and in combinations (*ITS*+*rbcL*, *matK*+*ITS*, *rbcL*+*matK*, *ITS*+*rbcL*+*matK*). The barcoding resolution of the single-locus DNA regions and multilocus barcodes was evaluated based on three different analytical methods i.e. the pair-wise genetic distance method (PWG-distance), the sequence similarity method (TaxonDNA) and phylogenetic methods (Neighbor-Joining trees, maximum likelihood and maximum parsimony trees).

The tested loci were examined in accordance with the CBOL guidelines for the distribution of intra- and inter-specific variability based on the PWG-distance (pairwise genetic distance) method. The PWG-distance was estimated by MEGA version 6 [41] using the Kimura two-parameter distance model (K2P) of nucleotide substitution with pairwise deletion of missing sites [42]. Average inter-specific distance was used to characterize inter-specific divergence [43, 44] and ‘all’ intra-specific distance, whilst mean ‘theta’ and coalescent depth were used to characterize intra-specific distances [26, 43]. Finally, the obtained inter- and intra-specific distances were plotted as a frequency distribution with a bin interval of 0.005 to illustrate the existing DNA barcoding gap [26]. In order to evaluate the local barcoding gap for each species, [43, 45] we plotted the maximum intra-specific divergences against the nearest neighbour (NN) distances for each species with a 1:1 slope [45, 46]. The total number of parsimony informative sites, mean GC content, variable number of nucleotides, i.e. the transition/transversion (T_s_/T_v_) ratio and the average length was obtained in MEGA6. In addition, the usefulness of the tested loci for barcoding was verified based on a direct comparison of sequence similarity; the proportion of correct identifications were annotated using Species Identifier 1.7.7 program from the TaxonDNA software package with ‘Best match’ (BM), ‘Best close match’ (BCM) and ‘All species barcodes’ functions [43]. The tool examines all the sequences present in an aligned data set and compares each successive sequence with all the other sequences to determine the closest match. The BM module then classifies the sequences as correct and incorrect based on whether the indicated pair is from a similar species, or a different species, respectively. While various equally good matches from different species are referred to as ambiguous in the BM module, the BCM module works on an intra-species variability criterion and is considered to be a more rigorous method in TaxonDNA. To test the accuracy of species assignments, the distributions of pairwise intra- and inter-specific distances for each candidate barcode with 0.005 distance intervals were also estimated in TaxonDNA with a ‘pairwise summary’ function using the K2P model, and the threshold was set to 95% [47].

The NJ and ML trees were constructed in PAUP 4.0 using K2P distances as the genetic measure and setting negative branch lengths to zero [48]. MP analysis was performed in PAUP 4.0 with the HKY-gamma substitution model to account for rate variation between sites. An initial heuristic search was made with 1000 replicates and branch swapping was performed by tree-bisection-reconnection (TBR). A maximum of 10 trees were held at each step with random stepwise addition for the starting tree in each replicate. The trees found in the first round were subjected to a second search by TBR swapping, holding up to 15000 trees and swapping to completion. The reliability of the node was assessed by a bootstrap test [49] with 1000 pseudo-replicates with the K2P distance options. Visualization and analysis of all the resulting trees was done in FigTree v1.4.2. [50, *http://tree.bio.ed.ac.uk/software/figtree*]. The methods were considered successful only when all the conspecific individuals formed a single clade with a bootstrap value above 60%.

### Database enrichment

Specimen data for each region were deposited in the Barcode of Life Data Systems (BOLD; http://www.boldsystems.org) under the project CRCBT–"DNA Barcoding in genus *Terminalia*". All the data are accessible online on BOLD under the dataset DS-TICIMAP. The sequences were submitted to GenBank (http://www.ncbi.nlm.nih.gov/genbank) and are publicly accessible under the accession numbers listed in S1 Table.

## Results

### PCR amplification, sequencing and genetic divergence

The sequence characteristics of all the tested barcoding regions are summarized in Table 2. All of the single region barcodes *rbcL*, *matK* and *ITS*, showed significant success rates (85–100%) for PCR amplification and sequencing reads using a single primer pair. On the contrary, *psbA-trnH* exhibited a low success rate, whereby 50% samples failed to generate high quality bidirectional sequences due to presence of a poly (T) tract at about 100bp from the *psbA* primer. Thus, *psbA-trnH* sequences were not acquired for a large number of species despite multiple attempts and analysis of the *psbA-trnH* region as a usable barcode in *Terminalia* was not pursued further.

**Table 2:** Summary of sequence characteristics of the different barcodes and their combinations used in this study.

Barcode locus/loci	No. of individuals	Length of sequences (bp)	Alignment range (bp)	No. of variable sites (%)	Ranges of intra-specific distances	Ranges of inter-specific distances	Mean intra-specific distance	Mean inter-specific distance
***rbcL***	90	633–636	636	22 (3.45)	0–0.0031	0–0.013	0.0003±0.0002	0.006±0.003
***matK***	80	525–531	598	240 (40.13)	0–0.4033	0–0.689	0.0144±0.0016	0.071±0.009
***ITS***	51	584–592	605	181 (29.91)	0–0.0660	0.010–0.124	0.0068±0.0011	0.076±0.012
***rbcL*+*matK***	62	1158–1167	1234	253 (20.50)	0–0.1276	0–0.222	0.0062±0.0007	0.033±0.004
***rbcL*+*ITS***	45	1220–1228	1241	178 (14.34)	0–0.2843	0.006–0.055	0.0035±0.0006	0.035±0.005
***matK*+*ITS***	36	1110–1121	1203	389 (32.33)	0–0.348	0.009–0.309	0.0188±0.0019	0.106±0.009
***rbcL*+*matK*+*ITS***	32	1746–1757	1839	391 (21.26)	0–0.820	0.009–0.176	0.01335±0.0012	0.069±0.006

**Fig 1:**
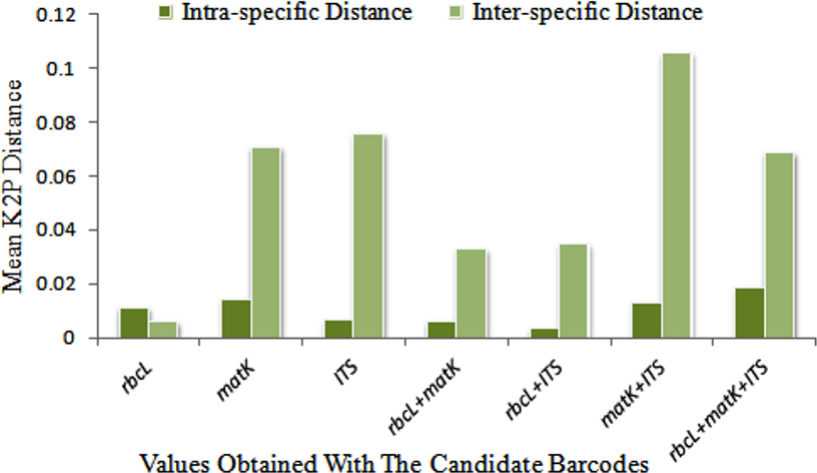
Mean intra- and inter-specific genetic distances of evaluated DNA regions based on the K2P model of nucleotide substitution. The x-axis reports the different DNA barcodes used either alone or in combination and the y-axis refers to mean K2P distance values.

The three tested regions viz. *rbcL*, *matK*, *and ITS* showed an optimal length (~ 525–636 bp) for barcode sequences. Overall, the aligned length ranged from 598 bp (*matK*) to 636 (*rbcL*) for a single locus and 1203 bp (*matK*+*ITS*) to 1839 bp (*rbcL*+*matK*+*ITS*) for combinations of loci. The alignment length for *ITS* region was 605 bp with 15 indels of 1–4 bp within the aligned region. The primers for ITS used in the study lie in the conserved flanking regions of 18S and 26S, so the sequences were trimmed to boundaries of the *ITS1*, *5*.*8S* and *ITS2 region*. *rbcL* was found to be highly conserved (95.9% identical sites) with the lowest number of variable sites (22/636), while *matK* and *ITS* showed the highest variability and length variation. Also, the percentage of identical pairwise residues including gap versus non-gap residues and excluding gap versus gap residues was highest in *rbcL* with 99.4% pairwise identity. Mean intra- and inter-specific genetic distances for all the tested regions based on pairwise distributions through MEGA are reported in Fig 1. *matK* exhibited the highest inter-specific sequence divergence (0.689) followed by its combination with either *ITS* (0.309) or *rbcL* (0.222). *rbcL* had the lowest inter-specific (0.013) and intra-specific (0.003) divergence. The highest intra-specific sequence distance was observed in the combination of regions *rbcL*+*matK*+*ITS* (0.820) followed by single locus *matK* (0.403). It is interesting to note that all the combinations that included *matK* demonstrated a greater range of inter-specific distance than the single regions, *rbcL* and *ITS*, alone, which renders *matK* most suitable to be the locus of choice in the genus *Terminalia* (Table 2).

### Species resolution gap and DNA barcoding assessment

**Fig 2:**
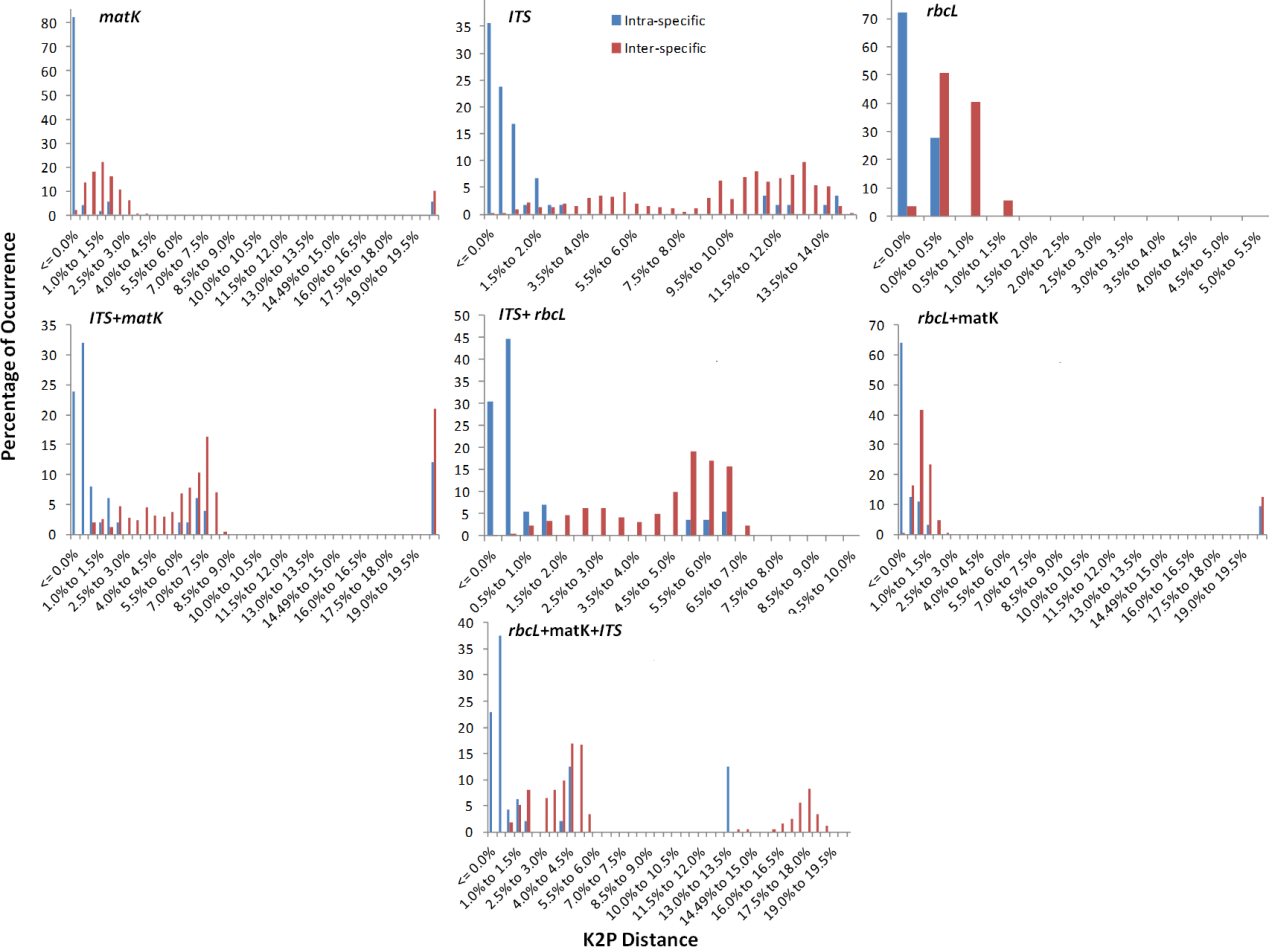
Relative distribution of intra-specific and inter-specific distances for all the single and combination of tested barcodes in *Terminalia*. x-axes denote K2P distances arranged in intervals, and the y-axes denote the percentage of occurrences.

**Fig 3:**
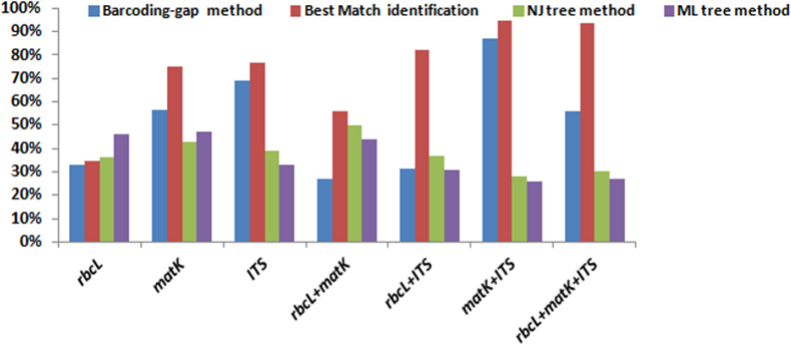
Identification success rates obtained using barcoding gap, best close match, NJ tree and ML tree methods for the DNA barcodes evaluated in this study.

**Fig 4:**
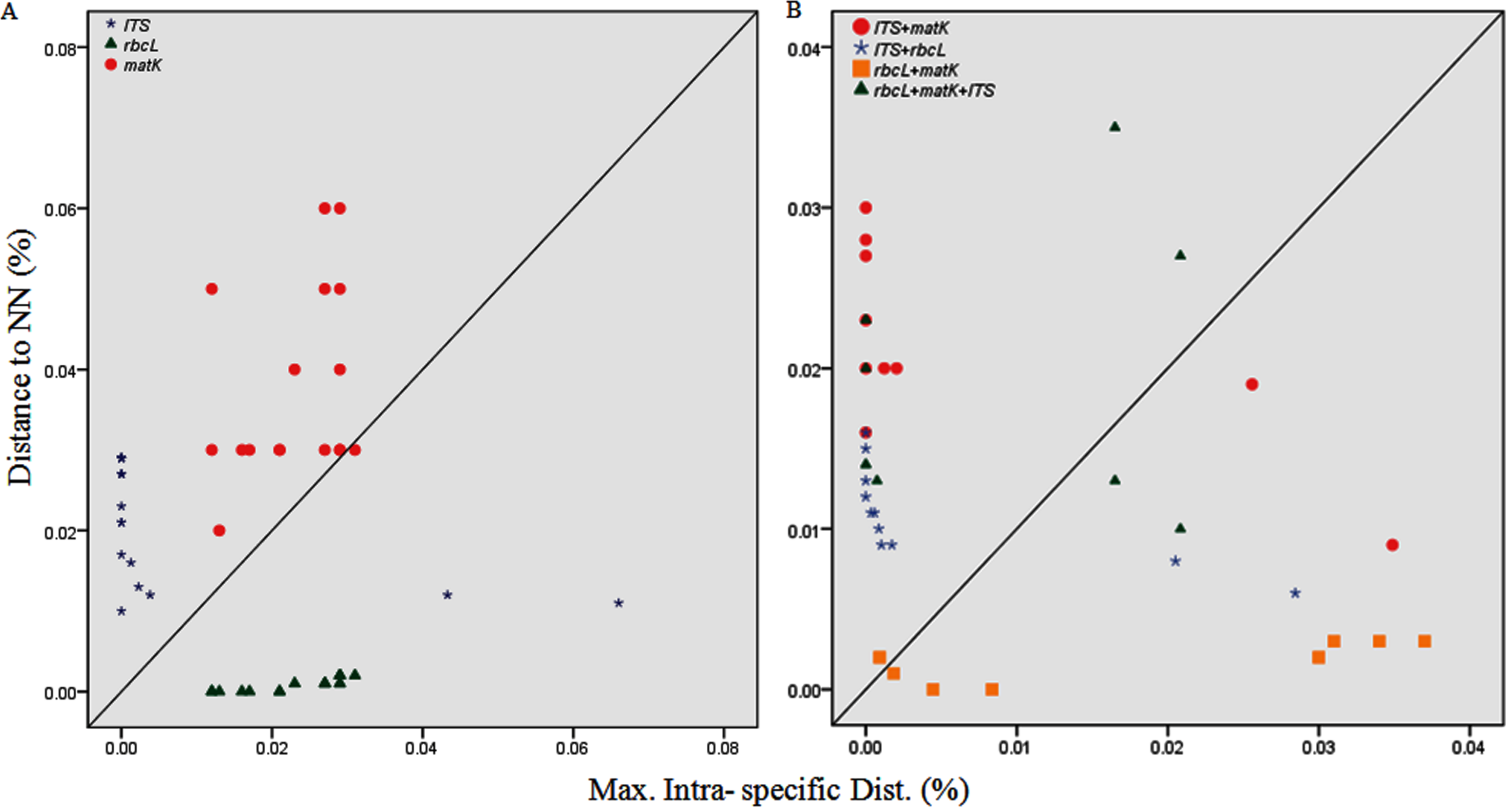
Barcode gap plot for the single and multilocus barcodes. Distance to nearest neighbour (NN) vs. maximum intra-specific K2P distances for the individual (A) and the combined (B) barcode regions. Each dot represents one or several individuals since they share identical values of intra-specific and inter-specific distances. Dots above the 1:1 line indicated the presence of a barcode gap. Due to the range of distances between analysed regions, the graphs presented are drawn to individual scales, according to the loci being compared.

Barcode gap analysis provides the distribution of distances within conspecifics and the distance to the nearest neighbour (NN) of each species. Comparison of the distance to the nearest non-conspecific versus the distance to the farthest conspecific revealed that the mean intra-specific distances were less than the distance to the nearest neighbour for each of the tested regions (Fig 1). Analysis of the relative distribution of K2P pairwise distances using TaxonDNA showed that each of the single loci *(rbcL*, *matK*, and *ITS*) lacked a global barcoding gap. On the other hand, the multilocus combinations *matK*+*ITS* (≈85%), *ITS*+*rbcL* (≈30%), *matK*+*rbcL* (≈25%) and *rbcL*+*matK*+*ITS* (≈55%) showed clear barcoding gaps in *Terminalia* species (Figs 2 and 3). The combination of *rbcL*+*matK*+*ITS* presented the greatest intra-specific variation (0–0.820) within the species (Table 2). The loci demonstrated clear barcoding gaps based on the frequency distribution of pairwise distances within the range 1.5% to 4.5%, though there is a slight overlap within and between species (Fig 2). The number of species with a barcoding gap indicates considerable sequence variation. The clear division between intra- and inter-specific sequence variations was further validated by local barcoding gap analysis in a 1:1 slope dot plot, which contrasted genetic distances within each species with the distance to its nearest neighbour (NN). Among the single regions, *matK* and *ITS* characterized most of the species (≈55–70%) above the line while *rbcL* exhibited very poor resolution (4A). The multilocus barcodes improved the percentage resolution and the combination of the *matK+ITS* regions showed the maximum resolution in presence of barcoding gap, by plotting all the species above the line with negligible exceptions (Fig 4B).

The BM and BCM analysis supported similar identification percentages for almost all the tested regions with very slight differences in the values. Based on the rigorousness of the method, results obtained from the BCM analysis are considered for further discussion. Both the BCM and All-species barcode modules of the SpeciesIdentifier program indicated the high efficiency of the *ITS* region (76.47% and 35.29% respectively) among the tested single locus barcodes (Table 3). The *matK* gene also showed close simililarity, with only a difference of 1.5–2.0%. The combination of *ITS* and *matK* was therefore concluded to be the best match with a 94.44% identification frequency, whilst *rbcL* presented the highest ambiguity among the sequences with an identification frequency of only 34.44%.

**Table 3:** Identification success rates based on analysis of the ‘best match’, ‘best close match’ and ‘all species barcodes’ function of TaxonDNA software for each DNA barcoding marker and its combinations.

Barcode locus/loci (No. of sequences)	Best close match*			All species barcode*		
	Correct (%)	Ambiguous (%)	Incorrect (%)	Correct (%)	Ambiguous (%)	Incorrect (%)
***rbcL* (90)**	34.44	**64.44**	1.11	27.77	**70.0**	2.22
***matK* (80)**	75.24	6.62	**18.13**	35.0	56.25	5.0
***ITS* (51)**	**76.47**	7.84	15.68	**35.29**	50.98	**13.72**
***rbcL*+*matK* (62)**	55.63	**34.23**	10.13	30.64	66.12	1.61
***rbcL*+*ITS* (45)**	82.22	4.44	**13.33**	**40.0**	48.88	**11.11**
***matK*+*ITS* (36)**	**94.44**	0.0	5.55	33.33	**66.66**	0.0
***rbcL*+*matK*+*ITS* (32)**	93.75	0.0	6.25	37.5	62.5	0.0

* Identification analysis was performed at 3% threshold. Highest values among the single and combination of barcodes for each function of the program are given in bold. The preferred barcoding option for identification of the *Terminalia* is highlighted in grey.

### Discrimination efficiency and taxonomic implications in *Terminalia*

**Fig 5:**
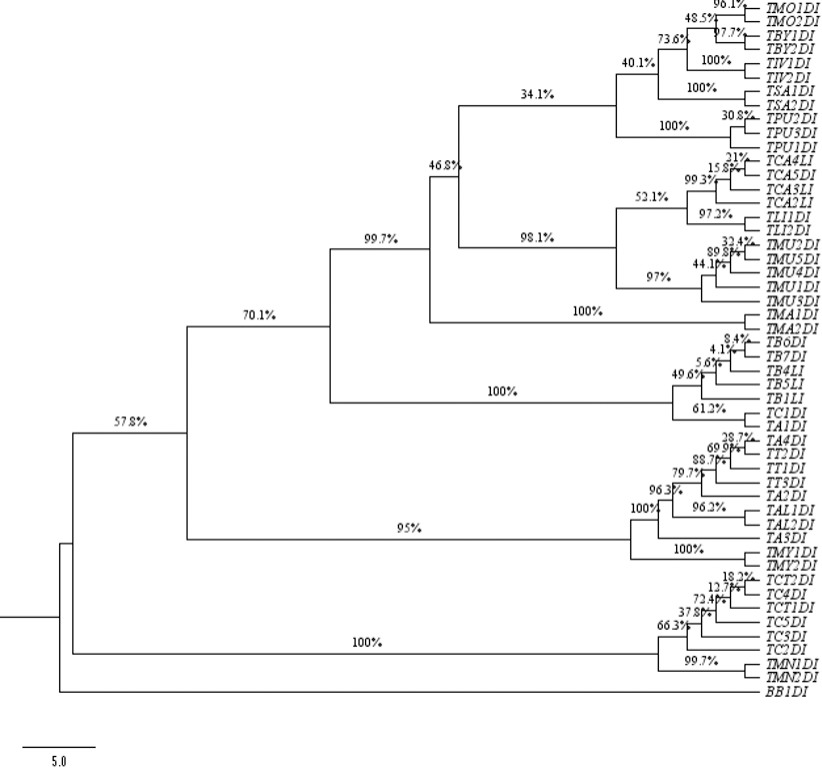
Neighbour-joining 50% majority-rule consensus tree using *ITS* DNA barcode.

**Fig 6:**
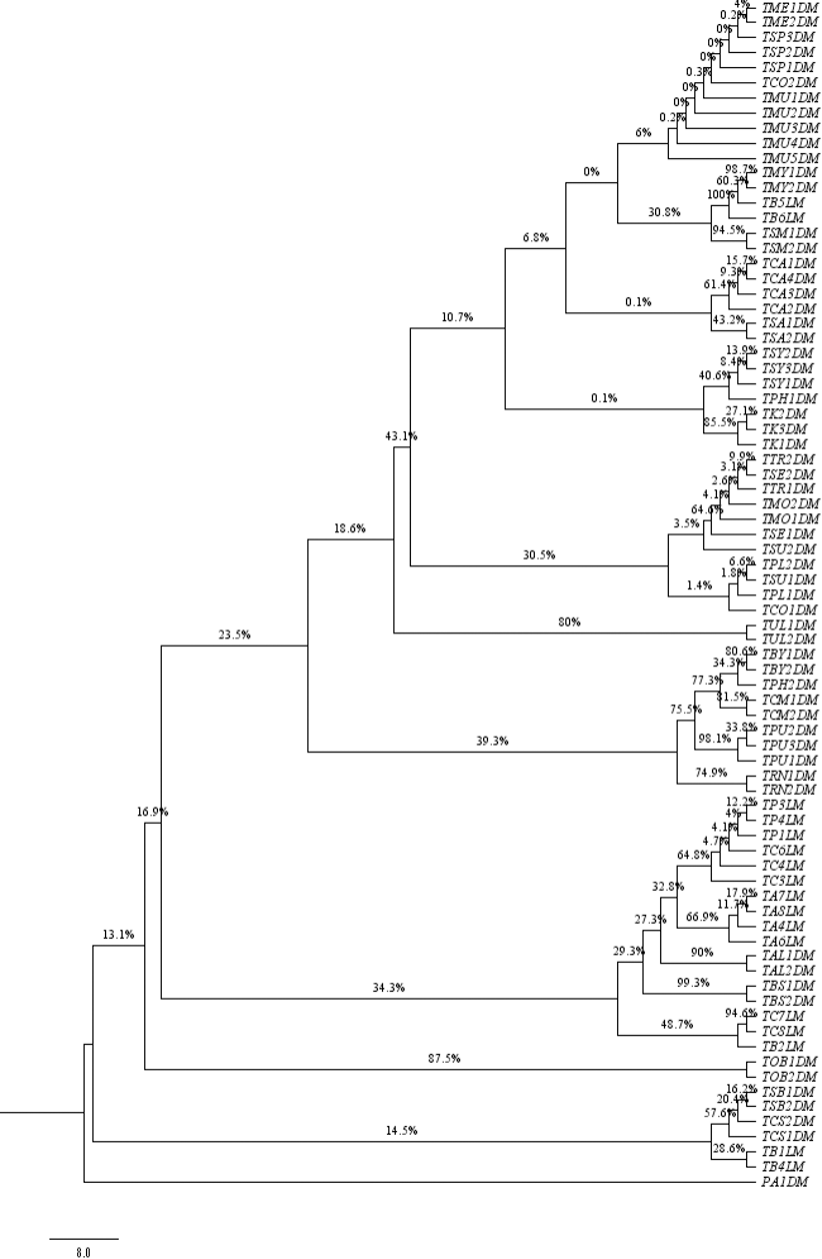
Neighbour-joining 50% majority-rule consensus tree using *matK* DNA barcode.

**Fig 7:**
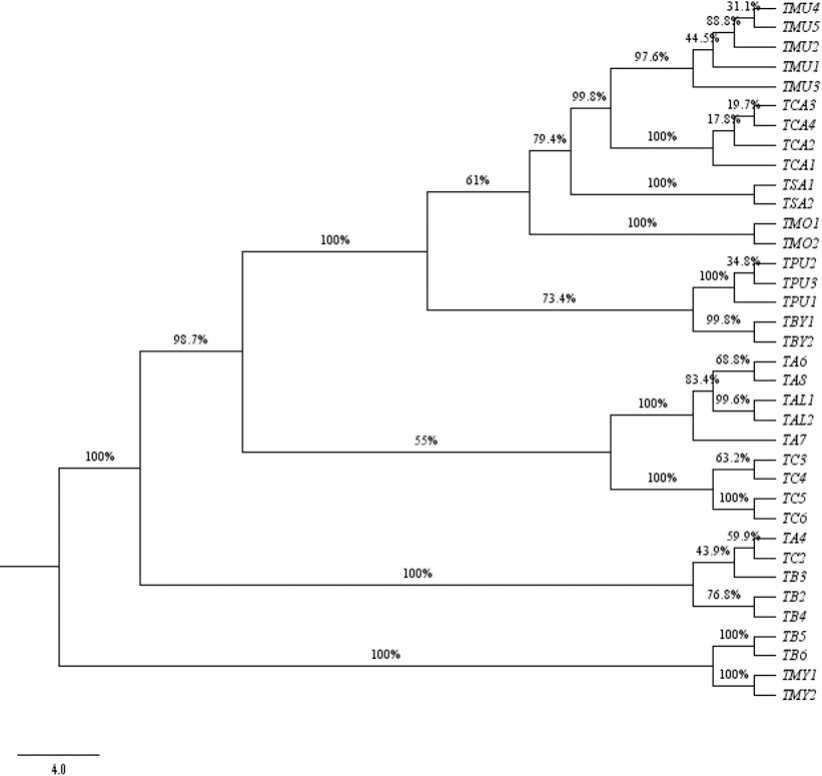
Strict consensus tree showing the relationship of *Terminalia* species resulting from Neighbour-joining analysis using the barcode *matK*+*ITS*.

**Fig 8:**
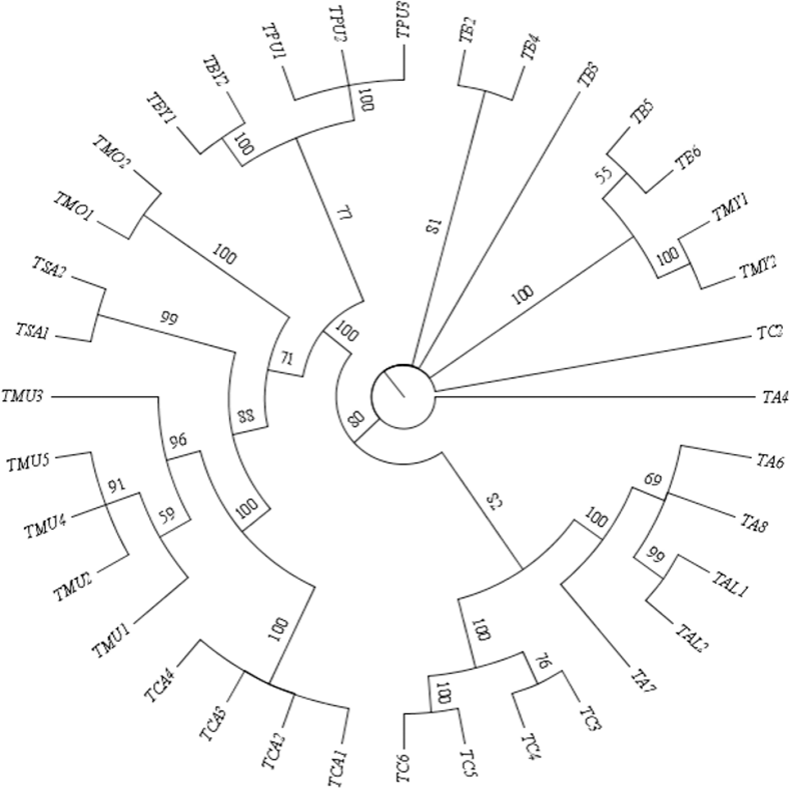
Maximum parsimony tree constructed using *matK*+*ITS* showing species resolution rates in terms of multi-locus barcode.

The analysis of barcode loci by computational phylogenetics displayed similar tree topologies, in agreement with results based on the presence of a barcoding gap. Amongst the three different phylogenetic tree methods tested, ML and NJ methods presented similar discriminatory results with reliable clade support. However, although the trees obtained through MP-based analysis showed similar clustering, they differed slightly in the percentage resolution (Table 4). Among the tested single region barcodes, *ITS* and *matK* identified 60–80% of the species, which was also found using barcoding gap technique (Figs 5 and 6). The relatively high success of identification was observed in the two locus combination *matK*+*ITS* (≈87.77%) using the NJ tree (Fig 7). The other two locus combinations, *rbcL*+*matK* and *rbcL*+*ITS*, showed 80.64% and 82.22% species resolution based on NJ analysis, respectively (Table 4). The most favoured barcode combination *matK*+*ITS* recorded 78% (CI = 0.7895) consistency index (CI) with the cladogram. The dataset for parsimony analysis included 1203 characters, of which 378 were parsimony informative and 11 variable characters were found to be parsimony-uninformative. The strict consensus tree of *matK*+*ITS* resulted in a tree length of 532 steps with the node supported clade framing the well resolved species of *Terminalia* (Fig 8). The retention index (RI) was 0.9312 and the re-scaled consistency index (RC) was 0.7352. Thus, the two-locus barcodes provided the highest identification accuracy among the regions tested, irrespective of the phylogenetic algorithms used in the study.

**Table 4:** The frequencies of correct identification (CI) for all samples based on sequence similarity method, pair-wise genetic distance method and tree-based analysis.

Barcode locus/loci (No. of Sequences)	CI Best close match (%)	DNA Barcoding gap (%)	Tree based (CI individuals) ^†^		
			NJ	ML	MP
***rbcL* (90)**	34.44%	33.0%	36	46	31
***matK* (80)**	75.24%	56.6%	43	47	39
***ITS* (51)**	76.47%	69.2%	39	33	39
***rbcL*+*matK* (62)**	55.63%	26.8%	50	44	28
***rbcL*+*ITS* (45)**	82.22%	31.5%	37	31	33
***matK*+*ITS* (36)**	94.44%	87.2%	28	26	26
***rbcL*+*matK*+*ITS* (32)**	93.75%	55.65%	30	27	27

^†^Based on the proportion of monophyletic species with 60% bootstrap.

Among all the studied *Terminalia* species, three species (*T*. *arjuna*, *T*. *chebula* and *T*. *bellerica*) showed the highest divergence in comparison to other monophyletic clades. *T*. *alata* bifurcated at the nodes from *T*. *arjuna* with 99.6% clade support. Both the species are morphologically characterized by the presence of 5 equal winged fruit as a differentiating character from the Cattapa section [7]. The combination of *matK+ITS* was able to resolve *T*. *catappa*, *T*. *chebula* and *T*. *bellerica* from the allied sections, which is in congruence with the fruit morphology in *Terminalia* (Fig 7). The other genera, *B*. *buceras*, *P*. *anisoptera*, and *A*. *acuminate*, taken as out-group in our study based on Maurin et al. 2010, were found to be embedded within the *Terminalia* species in the unrooted tree (Figures are available from the corresponding author). This finding indicates the complexity prevailing in the entire taxonomic level of Combretaceae [4]. To some extent, the relationship observed through molecular analysis based on single, and combinations of, barcodes were found to corroborate the traditional taxonomical framework. Most of the individuals belonging to *T*. *tomentosa* and *T*. *arjuna* were found to be intermixed within the clade in *ITS* region, which is in congruence with their taxonomical characters (Fig 5). The framing of single clusters from the representatives of most of the tested taxa allowed us to conclude that the investigated barcodes permitted successful molecular identification of species in the genus *Terminalia*.

## Discussion

### Discrimination success of various barcode candidates in *Terminalia* species

Hitherto, different analytical methods have been employed for the assessment of the species discrimination ability, including tree-based (NJ, MP, ML), distance-based (PWG-distance, p-distance, K2P-distance) and sequence similarity-based (BLAST and TaxonDNA) methods, with all of them differing in their discrimination power on the same data set [51–55]. In the present study, all the single region barcodes had a resolution ranging from 30–70%, which is much lower than the discriminatory rate of combined regions ≈95% (Fig 3). Thus, the single region barcode is not recommended for species discrimination in the genus *Terminalia*. Similar findings have been reported in previous studies in the family Combretaceae [4, 15]. In contrast, the recent phylogenetic study by Parani et al. 2016 [56], reports the highest suitability of the *psbA-trnH* intergenic spacer as a suitable barcode in *Terminalia*, but the findings are limited to the phylogenetic context. Our study indicates some negative features that discount the inclusion of *psbA-trnH* as a good barcode. The technical problems encountered in DNA sequencing and subsequent alignment ambiguities may be linked to mononucleotide repeats and insertion events, especially if they occur within species [57]. Consequently, this highlighted the need to search for multi-locus barcodes.

The present study concluded a very high identification efficiency for a combination of the ribosomal intergenic spacer *ITS* and the plastid coding gene *matK*, regardless of the method used. Both the barcodes demonstrated a higher range of inter-specific distances and lower range of intra-specific distances. The results of the best match method (94.44%), All-species barcode analysis (33.33%) and tree based analysis (~77.77%) proved the ability of the combination with data support. The species-specific monophyly demonstrated through tree-based analysis adds to its usefulness as a locus of choice in *Terminalia*. The greater discriminatory power at low taxonomic levels and higher evolutionary rate of nuclear region *ITS* makes it a promising locus in plant molecular systematics [58]. In addition, *matK* is one of the most rapidly evolving coding sections of plant genome with higher discrimination power than *rbcL* [59]. Prior barcoding studies published so far have also demonstrated the suitability of multiple regions with to improve the discriminatory power among closely related species, in line with the results obtained in the present study [23, 59–63]. Sequence analysis using TaxonDNA gave the highest species resolution based on the BM and BCM models, with either single or combinations of barcodes, followed by the tree-based NJ and ML methods. A similar pattern of result has been obtained from DNA barcoding studies in various plant groups [35].

The low efficiency of the *rbcL* gene observed in our study limits its usefulness in *Terminalia*, despite the utility of the region for DNA barcoding in other plant groups. The comparison of *rbcL* with other loci is sometimes found to be affected by the size of the dataset used in the study. It is to be noted that the set of specimens included in our *rbcL* analysis was comparatively large. Ren et al., 2010 [64] tested DNA barcoding to differentiate species of genus *Alnus*, and found *rbcL* to be the least performing locus with only 10% identification success. Similarly, Zhang et al., 2012 [63] had also reported the poor performance of *rbcL* as a locus of choice through their study in closely related groups of *Lysimachia* L. (Myrsinaceae). The two locus core barcode *rbcL+matK* had only 50% discrimination rate (Fig 3), which was lower than the identification rate of 72% proposed by the CBOL Plant Working Group (2009). The most plausible explanation for this discrepancy in *Terminalia* is the phylogenetically closely related taxa, which focused only on relative rather than absolute discrimination. Also the resolution rates of these two markers, alone and in combination have been concluded to decrease at the infrageneric level [15].

### Biological implications of DNA barcoding in *Terminalia*

The results obtained in the study shed some light on the phylogenetic classification of genus *Terminalia*, and are in agreement with the comprehensive study by Maurin et al. 2010 at the family level. About 93–95% of species based on NJ analysis, produced well supported clades with values above 60%. In *Terminalia* complex, many species (*T*. *arjuna*, *T*. *bellerica*, *T*. *chebula*, etc) have been found to exhibit significant within-species variation [7, 14, 65]. Worthy of special attention in this study is the fact that the phylogenetic constructions of the representatives from three potential species *T*. *arjuna*, *T*. *chebula* and *T*. *bellerica* showed high levels of divergence in their grouping. In particular, *Terminalia* species are predominantly outcrossing, and the sexual recombination and segregation along with geographical factors are the major sources of inter- as well as intra-specific variation in the entire genus [12]. The individuals sampled in the study were collected from a range of geographical localities and altitudes. Drastic differences in phenology between higher and lower altitudes and mountain barriers restrict the gene flow between the populations, resulting in complex and varied genetic variations.

Previous studies on the genus *Terminalia* had reported the complexity of species, which makes its taxonomic status highly problematic [4, 15, 56]. The genomic analysis of taxa with the barcode regions tested in this study provided molecular support for the taxonomic framework, exhibiting species monophyly for most of the species, with good posterior probability (Figs 7 and 8). *T*. *catappa* was found to be clustered with *T*. *muelleri* at 99.8% node support with all the individuals forming a monophyletic group. The polytomy of *T*. *sambesiaca* and *T*. *mollis* with *T*. *catappa* and *T*. *muelleri* is in agreement with recent molecular evidence [4]. We also noticed the grouping of *T*. *prunioides* and *T*. *brachystemma* at the base of one clade with 67.8% bootstrap value. *T*. *alata* was found to be well clustered between the individuals of *T*. *arjuna*, which is in line with morphological evidence. Both the species are characterized by the presence of five equal winged fruits. On the contrary, results for *T*. *bellerica* and *T*. *chebula* were found to be tangled with the representatives of *T*. *arjuna* based on the incongruent signal. Although the former two species share close morphological similarities, the latter is distinctly different.

The combination of morphological, ecological and reproductive biology with molecular data has paved a successful path for constructing a robust taxonomy for divergent plant taxa. Moreover, DNA barcoding is providing an opportunity to solve some taxonomic questions through discovering the underlying biological issues. Species discrimination for the genus *Terminalia* was high in biodiversity hotspots sampled in this study. Our results concluded that *ITS* in supplement with *matK* is the preferred choice for barcoding efforts aimed at the accurate identification for the species of *Terminalia*. The combinations of plastid and nuclear regions have shown to improve discriminatory power in many earlier barcoding studies [59, 66]. Its effectiveness can be determined in other systematic groups of plants through further studies. With the further exploration of species-specific single nucleotide polymorphisms (SNP), the present data will promote the progress of DNA barcoding in designing species-specific molecular markers for facilitating the targeted use of *Terminalia* species in trade and herbal market.

## Conclusions

The present study unequivocally demonstrates the efficiency of DNA barcoding in delimiting species boundaries in *Terminalia*. The signature sequences of the multilocus barcode *matK*+*ITS* (~77.77% through NJ) provided accurate markers for the molecular identity of species. The combined region showed a 87.2% barcoding gap among the species, which highlights its value in providing accurate species discrimination. The species from the Catappa section viz. *T*. *chebula*, *T*. *muelleri* and *T*. *bellerica* were well-resolved with supported results. Intra-specific divergence within the representatives of species *T*. *arjuna*, *T*. *chebula* and *T*. *bellerica* highlighted the effect of geographical factors. The correct identification frequency using the *matK*+*ITS* combination was 94.44% with the BCM module of TaxonDNA, which concludes it to be the best barcode in *Terminalia*. The efficiency of *ITS* at the species level indicates that it can be supplemented to the two core DNA chloroplast barcodes proposed by the CBOL plant working group, with further evaluation in other plant groups supporting its inclusion in a universal barcode for plants.

## Supporting information

S1 Table Sample details and GenBank (NCBI) accession numbers of for all the samples of *Terminalia* corresponding to different regions used in this study. Accessions numbers marked in bold represent the sequences generated in our lab.
(DOC)

S1 Text *rbcL* sequences alignment used in the study. (FASTA)

S2 Text *matK* sequences alignment used in the study. (FASTA)

S3 Text *ITS* sequences alignment used in the study. (FASTA)

S4 Text *rbcL*+*matK* sequences alignment (concatenated) used in the study. (FASTA)

S5 Text *ITS*+*rbcL* sequences alignment (concatenated) used in the study. (FASTA)

S6 Text *ITS*+*matK* sequences alignment (concatenated) used in the study. (FASTA)

S7 Text *rbcL*+*matK*+*ITS* sequences alignment (concatenated) used in the study. (FASTA)
