# Supplementary material for: Evaluation of single and multilocus DNA barcodes towards species delineation in complex tree genus Terminalia
Source: PLoS One. 2017 Aug 22;12(8):e0182836. doi: 10.1371/journal.pone.0182836 (PMC5567895; doi:10.1371/journal.pone.0182836)
Supplement: S1 Table — Accessions numbers marked in bold represent the sequences generated in our lab. (DOC) [file pone.0182836.s001.doc]

**S1 Table: Sample details and GenBank (NCBI) accession numbers of for all the samples of *Terminalia* corresponding to different regions used in this study. Accessions numbers marked in bold represent the sequences generated in our lab.**

| **Taxon** | **Region** | **GenBank (NCBI) Accessions No.** |
| --- | --- | --- |
| *Terminalia arjuna* | *rbcL* | **KT274008.1** |
| *Terminalia arjuna* | *rbcL* | **KT279738.1** |
| *Terminalia arjuna* | *rbcL* | **KT279739.1** |
| *Terminalia bellirica* | *rbcL* | **KT274009.1** |
| *Terminalia bellirica* | *rbcL* | **KT274010.1** |
| *Terminalia bellirica* | *rbcL* | **KT279740.1** |
| *Terminalia catappa* | *rbcL* | **KT274011.1** |
| *Terminalia catappa* | *rbcL* | **KT274012.1** |
| *Terminalia catappa* | *rbcL* | **KT279741.1** |
| *Terminalia chebula* | *rbcL* | **KT203920.1** |
| *Terminalia chebula* | *rbcL* | **KT203921.1** |
| *Terminalia chebula* | *rbcL* | **KT203922.1** |
| *Terminalia paniculata* | *rbcL* | **KT274014.1** |
| *Terminalia paniculata* | *rbcL* | **KT274015.1** |
| *Terminalia paniculata* | *rbcL* | **KT279742.1** |
| *Terminalia arjuna* | *rbcL* | FJ381821.1 |
| *Terminalia muelleri* | *rbcL* | AF425712.1 |
| *Terminalia tomentosa* | *rbcL* | FJ381819.1 |
| *Terminalia myriocarpa* | *rbcL* | FJ381816.1 |
| *Terminalia chebula* | *rbcL* | AF425710.1 |
| *Terminalia chebula* | *rbcL* | FJ381812.1 |
| *Terminalia muelleri* | *rbcL* | AF425712.1 |
| *Terminalia bellirica* | *rbcL* | FJ381808.1 |
| *Terminalia tomentosa* | *rbcL* | FJ381819.1 |
| *Terminalia trichopoda* | *rbcL* | FJ381820.1 |
| *Terminalia mollis* | *rbcL* | EU338118.1 |
| *Terminalia sambesiaca* | *rbcL* | FJ381817.1 |
| *Terminalia phanerophlebia* | *rbcL* | EU338119.1 |
| *Terminalia mantaly* | *rbcL* | FJ381815.1 |
| *Terminalia ivorensis* | *rbcL* | FJ381813.1 |
| *Terminalia litoralis* | *rbcL* | FJ381814.1 |
| *Terminalia sericea* | *rbcL* | EU338121.1 |
| *Terminalia amazonia* | *rbcL* | KU761915.1 |
| *Terminalia catappa* | *rbcL* | FJ381811.1 |
| *Terminalia bucidoides* | *rbcL* | KU761916.1 |
| *Terminalia ulexoides* | *rbcL* | KU761920.1 |
| *Terminalia ivorensis* | *rbcL* | KU761918.1 |
| *Terminalia mucronata* | *rbcL* | LC107040.1 |
| *Terminalia* *muelleri* | *rbcL* | AF425712.1 |
| *Terminalia prunioides* | *rbcL* | EU338120.1 |
| *Terminalia citrina* | *rbcL* | LC107030.1 |
| *Terminalia kaernbachii* | *rbcL* | KU761919.1 |
| *Terminalia brassii* | *rbcL* | KF753938.1 |
| *Terminalia franchetii* | *rbcL* | KF753936.1 |
| *Terminalia mantaly* | *rbcL* | LC107042.1 |
| *Terminalia subspathulata* | *rbcL* | KF753917.1 |
| *Terminalia bucidoides* | *rbcL* | KU761916.1 |
| *Terminalia* *muelleri* | *rbcL* | AF425712.1 |
| *Terminalia kaernbachii* | *rbcL* | KF753930.1 |
| *Terminalia kaernbachii* | *rbcL* | KF753930.1 |
| *Terminalia superba* | *rbcL* | KF753920.1 |
| *Terminalia oblonga* | *rbcL* | KF753915.1 |
| *Terminalia phellocarpa* | *rbcL* | KF753934.1 |
| *Terminalia nitens* | *rbcL* | KF753933.1 |
| *Terminalia samoensis* | *rbcL* | KF753921.1 |
| *Terminalia complanata* | *rbcL* | KF753918.1 |
| *Terminalia calamansanay* | *rbcL* | KF753914.1 |
| *Terminalia melanocarpa* | *rbcL* | KF753931.1 |
| *Terminalia glaucescens* | *rbcL* | KF753929.1 |
| *Terminalia ulexoides* | *rbcL* | KF753913.1 |
| *Terminalia bellirica* | *rbcL* | KR530130.1 |
| *Terminalia bucidoides* | *rbcL* | KF753923.1 |
| *Anogeissus acuminata* | *rbcL* | AF425708.1 |
| *Terminalia prunioides* | *rbcL* | EU338120.1 |
| *Terminalia tomentosa* | *rbcL* | FJ381819.1 |
| *Terminalia myriocarpa* | *rbcL* | FJ381816.1 |
| *Terminalia* *muelleri* | *rbcL* | AF425712.1 |
| *Terminalia trichopoda* | *rbcL* | FJ381820.1 |
| *Terminalia mollis* | *rbcL* | EU338118.1 |
| *Terminalia sambesiaca* | *rbcL* | FJ381817.1 |
| *Terminalia phanerophlebia* | *rbcL* | EU338119.1 |
| *Terminalia litoralis* | *rbcL* | FJ381814.1 |
| *Terminalia amazonia* | *rbcL* | KU761915.1 |
| *Terminalia glaucescens* | *rbcL* | KF753929.1 |
| *Terminalia mucronata* | *rbcL* | LC107040.1 |
| *Terminalia prunioides* | *rbcL* | EU338120.1 |
| *Terminalia citrina* | *rbcL* | LC107030.1 |
| *Terminalia brassii* | *rbcL* | KF753938.1 |
| *Terminalia franchetii* | *rbcL* | KF753936.1 |
| *Terminalia subspathulata* | *rbcL* | KF753917.1 |
| *Terminalia superba* | *rbcL* | KF753920.1 |
| *Terminalia oblonga* | *rbcL* | KF753915.1 |
| *Terminalia phellocarpa* | *rbcL* | KF753934.1 |
| *Terminalia nitens* | *rbcL* | KF753933.1 |
| *Terminalia samoensis* | *rbcL* | KF753921.1 |
| *Terminalia complanata* | *rbcL* | KF753918.1 |
| *Terminalia calamansanay* | *rbcL* | KF753914.1 |
| *Terminalia melanocarpa* | *rbcL* | KF753931.1 |
| *Terminalia sericea* | *rbcL* | EU338121.1 |
| *Anogeissus acuminata* | *rbcL* | AF425708.1 |
| *Terminalia arjuna* | *matK* | **KT274007.1** |
| *Terminalia arjuna* | *matK* | **KT279716.1** |
| *Terminalia arjuna* | *matK* | **KT279717.1** |
| *Terminalia bellirica* | *matK* | **KT274002.1** |
| *Terminalia bellirica* | *matK* | **KT274003.1** |
| *Terminalia bellirica* | *matK* | **KT279718.1** |
| *Terminalia chebula* | *matK* | **KT274004.1** |
| *Terminalia chebula* | *matK* | **KT274005.1** |
| *Terminalia chebula* | *matK* | **KT279719.1** |
| *Terminalia paniculata* | *matK* | **KT274006.1** |
| *Terminalia paniculata* | *matK* | **KT279720.1** |
| *Terminalia paniculata* | *matK* | **KT279721.1** |
| *Terminalia arjuna* | *matK* | JX495766.1 |
| *Terminalia alata* | *matK* | AB925073.1 |
| *Terminalia complanata* | *matK* | GQ248208.1 |
| *Terminalia calamansanay* | *matK* | AB924847.1 |
| *Terminalia phellocarpa* | *matK* | KF793975.1 |
| *Terminalia ulexoides* | *matK* | KF793958.1 |
| *Terminalia complanata* | *matK* | KF793962.1 |
| *Terminalia stenostachya* | *matK* | KC130271.1 |
| *Terminalia mollis* | *matK* | JX518150.1 |
| *Terminalia prunioides* | *matK* | JF270967.1 |
| *Terminalia catappa* | *matK* | JX518026.1 |
| *Terminalia trichopoda* | *matK* | JX517390.1 |
| *Terminalia phanerophlebia* | *matK* | JF270966.1 |
| *Terminalia chebula* | *matK* | AB924845.1 |
| *Terminalia sambesiaca* | *matK* | JX517421.1 |
| *Terminalia bellirica* | *matK* | KR531558.1 |
| *Terminalia stenostachya* | *matK* | KC130271.1 |
| *Terminalia sepicana* | *matK* | KF793957.1 |
| *Terminalia circumalata* | *matK* | KF793969.1 |
| *Terminalia brassii* | *matK* | KF793977.1 |
| *Terminalia brachystemma* | *matK* | JX518028.1 |
| *Terminalia phanerophlebia* | *matK* | JX517994.1 |
| *Terminalia prunioides* | *matK* | JF270967.1 |
| *Terminalia muelleri* | *matK* | GU135121.1 |
| *Terminalia sericea* | *matK* | JX517972.1 |
| *Terminalia melanocarpa* | *matK* | KF793973.1 |
| *Terminalia catappa* | *matK* | GU135057.1 |
| *Terminalia kaernbachii* | *matK* | KF793972.1 |
| *Terminalia calamansanay* | *matK* | KF793959.1 |
| *Terminalia myriocarpa* | *matK* | KR531564.1 |
| *Terminalia muelleri* | *matK* | GU135121.1 |
| *Terminalia samoensis* | *matK* | KF793964.1 |
| *Terminalia mollis* | *matK* | KC130314.1 |
| *Terminalia subspathulata* | *matK* | KF793961.1 |
| *Terminalia oblonga* | *matK* | KF793960.1 |
| *Terminalia sericea* | *matK* | JF270968.1 |
| *Terminalia muelleri* | *matK* | GU135121.1 |
| *Terminalia randii* | *matK* | JX518067.1 |
| *Terminalia stenostachya* | *matK* | JX517373.1 |
| *Terminalia bellirica* | *matK* | KR531562.1 |
| *Terminalia muelleri* | *matK* | GU135121.1 |
| *Terminalia prunioides* | *matK* | JF270967.1 |
| *Terminalia superba* | *matK* | KF793963.1 |
| *Terminalia kaernbachii* | *matK* | KF793972.1 |
| *Pteleopsis anisoptera* | *matK* | JX517605.1 |
| *Terminalia catappa* | *matK* | GU135057.1 |
| *Terminalia catappa* | *matK* | JX518026.1 |
| *Terminalia chebula* | *matK* | AB924845.1 |
| *Terminalia myriocarpa* | *matK* | KR531564.1 |
| *Terminalia muelleri* | *matK* | GU135121.1 |
| *Terminalia trichopoda* | *matK* | JX517390.1 |
| *Terminalia brachystemma* | *matK* | JX518028.1 |
| *Terminalia sambesiaca* | *matK* | JX517421.1 |
| *Terminalia ulexoides* | *matK* | KF793958.1 |
| *Terminalia kaernbachii* | *matK* | KF793972.1 |
| *Terminalia brassii* | *matK* | KF793977.1 |
| *Terminalia subspathulata* | *matK* | KF793961.1 |
| *Terminalia superba* | *matK* | KF793963.1 |
| *Terminalia oblonga* | *matK* | KF793960.1 |
| *Terminalia phellocarpa* | *matK* | KF793975.1 |
| *Terminalia samoensis* | *matK* | KF793964.1 |
| *Terminalia melanocarpa* | *matK* | KF793973.1 |
| *Terminalia alata* | *matK* | AB925073.1 |
| *Terminalia sepicana* | *matK* | KF793957.1 |
| *Terminalia circumalata* | *matK* | KF793969.1 |
| *Terminalia randii* | *matK* | JX518067.1 |
| *Pteleopsis anisoptera* | *matK* | JX517605.1 |
| *Terminalia bellirica* | *ITS* | **KT235565.1** |
| *Terminalia bellirica* | *ITS* | **KT279734.1** |
| *Terminalia bellirica* | *ITS* | **KT279735.1** |
| *Terminalia catappa* | *ITS* | **KT235566.1** |
| *Terminalia catappa* | *ITS* | **KT279736.1** |
| *Terminalia catappa* | *ITS* | **KT279737.1** |
| *Terminalia bellirica* | *ITS* | FJ381773.1 |
| *Terminalia bellirica* | *ITS* | KC602394.1 |
| *Terminalia chebula* | *ITS* | KC984654.1 |
| *Terminalia arjuna* | *ITS* | FM887017.1 |
| *Terminalia mantaly* | *ITS* | FJ381778.1 |
| *Terminalia litoralis* | *ITS* | FJ381777.1 |
| *Terminalia ivorensis* | *ITS* | FJ381776.1 |
| *Terminalia sambesiaca* | *ITS* | FJ381780.1 |
| *Terminalia sambesiaca* | *ITS* | JX840498.1 |
| *Terminalia ivorensis* | *ITS* | FJ381776.1 |
| *Terminalia brachystemma* | *ITS* | JX840550.1 |
| *Terminalia mollis* | *ITS* | EU338008.1 |
| *Terminalia mantaly* | *ITS* | LC050569.1 |
| *Terminalia chebula* | *ITS* | FJ381775.1 |
| *Terminalia prunioides* | *ITS* | EU338010.1 |
| *Terminalia chebula* | *ITS* | HM236857.1 |
| *Terminalia catappa* | *ITS* | LC050568.1 |
| *Terminalia chebula* | *ITS* | LC050566.1 |
| *Terminalia myriocarpa* | *ITS* | FJ381779.1 |
| *Terminalia mucronata* | *ITS* | LC050563.1 |
| *Terminalia citrina* | *ITS* | LC050564.1 |
| *Terminalia brachystemma* | *ITS* | FJ381774.1 |
| *Terminalia chebula* | *ITS* | LC050565.1 |
| *Terminalia muelleri* | *ITS* | JX856525.1 |
| *Terminalia muelleri* | *ITS* | JX856524.1 |
| *Terminalia muelleri* | *ITS* | JX856523.1 |
| *Terminalia tomentosa* | *ITS* | FJ381781.1 |
| *Bucida buceras* | *ITS* | FJ381771.1 |
| *Terminalia muelleri* | *ITS* | AF334767.1 |
| *Terminalia mucronata* | *ITS* | LC050563.1 |
| *Terminalia tomentosa* | *ITS* | KR425506.1 |
| *Terminalia muelleri* | *ITS* | AF160472.1 |
| *Terminalia arjuna* | *ITS* | FJ381783.1 |
| *Terminalia alata* | *ITS* | LC050570.1 |
| *Terminalia citrina* | *ITS* | LC050564.1 |
| *Terminalia tomentosa* | *ITS* | KT187391.1 |
| *Terminalia alata* | *ITS* | LC050570.1 |
| *Terminalia arjuna* | *ITS* | KF925432.1 |
| *Terminalia arjuna* | *ITS* | AF338255.1 |
| *Terminalia prunioides* | *ITS* | JX840533.1 |
| *Terminalia mollis* | *ITS* | JX840493.1 |
| *Terminalia circumalata* | *ITS* | KF794091.1 |
| *Terminalia prunioides* | *ITS* | JX840591.1 |
| *Terminalia myriocarpa* | *ITS* | KR532657.1 |
| *Terminalia circumalata* | *ITS* | KF794091.1 |
| *Terminalia litoralis* | *ITS* | FJ381777.1 |
| *Bucida buceras* | *ITS* | FJ381771.1 |
